# Combined liver-kidney transplant in polycystic diseases: a case report

**DOI:** 10.31744/einstein_journal/2023RC0282

**Published:** 2023-05-23

**Authors:** Olival Cirilo Lucena da Fonseca, Beatriz Costa Nava Martins, Norma Thomé Jucá, Victor Cruz Rosa Alencar de Sá, Priscylla Jennie Monteiro Rabêlo, Paulo Sérgio Vieira de Melo, Américo Gusmão Amorim, Cláudio Moura Lacerda

**Affiliations:** 1 Hospital Universitário Oswaldo Cruz Recife PE Brazil Unidade de Transplante de Fígado, Hospital Universitário Oswaldo Cruz, Recife, PE, Brazil.; 2 Faculdade de Ciências Médicas Universidade de Pernambuco Recife PE Brazil Faculdade de Ciências Médicas, Universidade de Pernambuco, Recife, PE, Brazil.; 3 Instituto de Medicina Integral Professor Fernando Figueira Recife PE Brazil Instituto de Medicina Integral Professor Fernando Figueira, Recife, PE, Brazil.

**Keywords:** Hepatomegaly, Polycystic kidney, autosomal dominant, Liver failure, Liver diseases, Liver transplantation, Kidney transplantation

## Abstract

Polycystic liver disease, a hereditary pathology, usually manifests as autosomal dominant polycystic kidney disease. The many cysts in the liver cause massive hepatomegaly, majorly affecting the patient’s quality of life. In cases of refractory symptoms, liver transplantation is the only treatment choice. A 43-year-old woman was followed up as a hepatology outpatient in August 2020, with a progressive increase in abdominal volume, lower limb edema, and cachexia. The patient was diagnosed with polycystic renal and liver disease with massive hepatomegaly in March 2021, a combined kidney-liver transplant. Liver size represented 13% of the patient’s corporal composition, weighing 8.6kg. The patient was discharged on the 7^th^ postoperative day with no complications. Only 10-20% of patients with polycystic liver disease have clinical manifestations, most of which result from hepatomegaly. An increase in liver volume deteriorates liver function until the condition becomes end-stage liver disease, as kidney function is already compromised; liver-kidney transplantation remains the only treatment choice. The case described drew significant attention to the massive hepatomegaly presented in the patient, with the liver representing over 10% of the patient’s body weight, approximately five to six times larger than a normal-sized liver.

## INTRODUCTION

Polycystic liver disease (PLD) is a hereditary pathology usually associated with autosomal dominant polycystic kidney disease (ADPKD).^( 1 )^ Most clinical manifestations result from multiple hepatic cysts; increased liver volume, associated with organ impairment, causes systematic repercussions from reflux and dyspnea due to esophageal and diaphragmatic compression until end-stage liver disease symptoms.^( 2 )^

While clinical treatment is typically the initial choice for patients with polycystic liver disease and related symptoms, liver transplantation is often the preferred treatment option. This is particularly true when liver disease is accompanied by hepatomegaly and stubborn symptoms. If renal failure is also present, double transplantation, involving both the liver and kidney, is often necessary.^( 3 )^ We describe a case of a patient submitted to a double transplant due to renal and hepatic polycystic disease with a liver weighing 8.6kg - 13% of the patient body composition.

## CASE REPORT

In August 2020, a 43-year-old woman was followed up in a hepatology ambulatory at *Hospital Universitário Oswaldo Cruz* , Recife, Brazil, with a progressive increase in abdominal volume, lower limb edema, and cachexia. The patient also had systemic arterial hypertension and a family history of liver disease. Upon physical examination, she presented with a globose abdomen-palpable liver 10cm above the xiphoid process, massive ascites, and noticeable lower-limb edema.

An abdominal ultrasound revealed an enlarged liver, with multiple cysts occupying most of the parenchyma, along with kidneys in a similar situation: multiple cysts, characterizing, then, polycystic kidney and liver disease. Abdominal tomography revealed massive hepatomegaly and bilateral nephromegaly with many cysts of different dimensions, compatible with the clinical picture of the patient’s hepatic and renal impairment.

We prescribed diuretics; however, the lower limb edema and abdominal volume worsened. In October 2020, the patient developed refractory ascites, troubling edema, and decay in nitrogenous slag, requiring multiple hospitalizations for dialysis and paracentesis. Due to refractory ascites, persistent edema, and other clinical symptoms that cause loss of life quality, a decrease in liver function, with INR = 1,5, bilirubin = 5,0, MELD = 25, associated with chronic kidney impairment, performing hemodialysis 3 days per week and creatinine = 7,66, the medical team indicated a combined liver-kidney transplant. She had been undergoing hemodialysis for 7 months while on the transplant waitlist.

In March 2021, a compatible donor was identified. A 35-year-old male who died from traumatic brain injuries was classified as a marginal donor because of the following risk factors: high doses of vasoactive drugs and hospitalization longer than 4 days in the intensive care unit.

**Figure 1:**
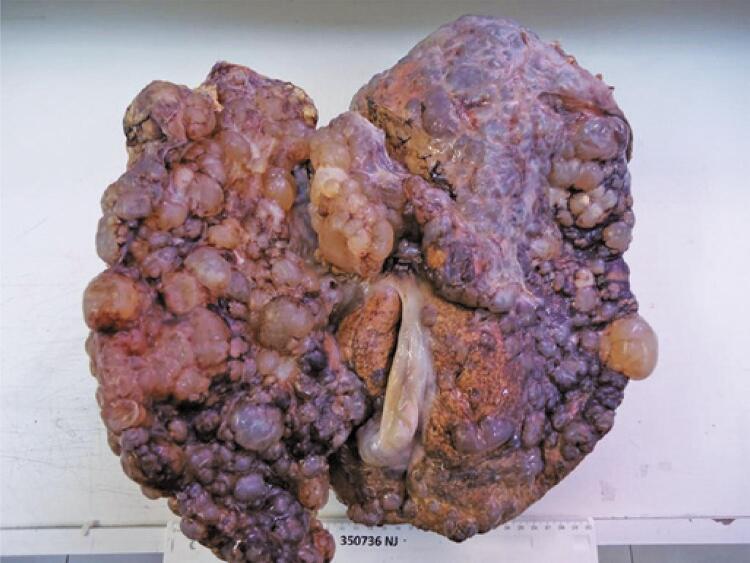
Surgical specimen – liver parenchyma replaced by multiple cysts

**Figure 2:**
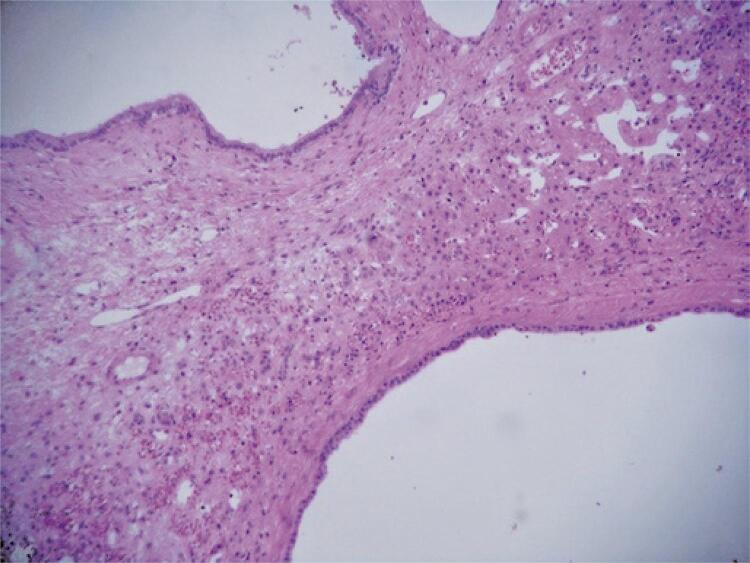
Anatomopathological examination

The patient was admitted with a weight of 65.6kg, body mass index of 24kg/m^2^, MELD of 25, MELD-Na of 26, and Child-Pugh B. The transplant technique was piggy-back with retrograde reperfusion and the usual portal, arterial, and biliary reconstructions. After opening the abdominal cavity, we drained 7L of ascites, followed by liver dissection, which was a considerable challenge owing to the organ’s size, shape, and weight. The liver transplant lasted for 6 hours and 11 minutes, respectively. During the procedure, 7L of electrolyte solution, as well as three bottles of albumin and 894mL of red blood cells, were infused. Following that, another surgical team performed renal transplantation with the removal of both native kidneys, with transient hypoperfusion of moderate intensity and small wall extension, 12 hours ischemia time, with a satisfactory conclusion. The kidney and liver donors were the same. Kidney transplantation lasted 3 hours and 40 minutes. The patient evolves in the postoperative without complications, with adequate renal function and diuresis on the first day. She was discharged on the 7^th^ day with tacrolimus, mycophenolate, and prednisone. The surgical specimen measured 38.0 x 30.0 x 14.0cm and weighed 8,600g. Liver histopathology showed parenchyma replaced by multiple cysts, sinusoidal congestion, fibrocystic disease, and hepatic impairment ( Figures 1 and 2 ).

This study was approved by the Research Ethics of *Hospital Universitário Oswaldo Cruz* under CAAE: 60541622.7.0000.5192; # 5.591.212.

## DISCUSSION

Polycystic liver disease (PCOS) is a hereditary disease characterized by multiple cysts in the liver parenchyma. The Gigot classification is currently used for disease severity categorization. Polycystic liver disease can be classified as isolated polycystic liver disease, PLD as part of ADPKD, or PLD as part of ARPKD. Autosomal dominant polycystic kidney disease is the most common presentation of this disease. More than half of patients with ADPKD have cysts associated with the liver.^( 1 , 4 )^

Only 10-20% of patients have clinical manifestations resulting from hepatomegaly due to multiple cysts.^( 2 , 3 , 5 )^ The most common symptoms of ADPKD in the kidneys are hypertension, flank pain, and urinary tract complications. Approximately half of the patients with ADPKD have chronic renal failure and are indicated for transplantation.^( 6 )^ Liver volume also affects the patient’s prognosis; worsening liver function associated with repeated hospitalization and refractory symptoms strongly affects the patient’s quality of life.^( 7 )^Our patient presented with poor nutrition, esophageal and gastric symptoms such as dyspnea and reflux, massive ascites, and back pain.^( 2 , 3 , 5 )^ Some risk factors for the disease include the feminine gender, advanced age, and multiple pregnancies.^( 8 )^

Most patients require clinical treatment, such as drug therapy, focusing on improving symptoms and complications, such as hypertension, urinary tract infections, ascites, and edema. Percutaneous therapy with the aspiration of some cysts is indicated when the cyst is at least 5cm and contributes to the patient’s clinical deterioration. A surgical approach is necessary in cases with refractory symptoms and loss of quality of life. There were no objective indications for liver transplantation, and a specialist analyzed each case. Surgery is the only choice to cure the disease, and it is usually indicated for patients who do not improve with other therapies and those with progressive liver impairment.^( 1 , 2 , 4 )^

Double transplantation is generally indicated in end-stage liver disease and irreversible kidney disease patients. However, not all cases are subject to double transportation for the following reasons: lower morbidity and mortality, lower health costs, protection against the stage of terminal illness, etc. Although the number of double transplantations has increased in recent years, there are no objective guidelines for the time and indication for the procedure. The absence of standardized protocols and severity criteria impairs our ability to define who and when to indicate.^( 9 )^

An important data to be considered in patients on the waiting list is the prediction of irreversible or progressive kidney disease, especially in those already undergoing dialysis. Another point that must be evaluated is the presence of hepatorenal syndrome, which worsens the prognosis. Objectively, the patient’s general condition, graft availability, and the extent and severity of renal function should be evaluated. There are no clear indication criteria based only on organ size data, but these must be considered due to the impact on the patient’s life.^( 9 )^

Although liver transplantation is the only way to cure this disease, the indication for the procedure is due to the clinical burden posed by the liver, not because of the classical type of cirrhosis. Therefore, it is important to balance the real need to subject healthy patients to immunosuppression for the rest of life.^( 10 )^

Regarding the technique used in this case of liver transplantation, half of the studies applied portal bypass, and the other half applied piggy-back. None of the two techniques demonstrated superiority, with the choice of resting solely on the intraoperative conditions in the dissection phase. The portal bypass technique was chosen due to access to the suprahepatic vena cava and portal clamping.^( 11 )^

Double kidney-liver transplantation promotes excellent results and survival rates when the procedure is well-indicated, but this indication is not so common. A study that evaluated the survival of four double transplants showed a survival rate >70% two years after the procedure. When we compared the success rate of procedure indications, such as hepatocellular carcinoma and chronic liver failure, in PLD, the survival rate was higher than the others. Two studies showed a survival rate of 85-90% during the first 5 years after liver transplantation. The European liver transplantation group showed a 1-year survival rate of nearly 90%, a 5-year survival rate of 85%, and a 10-year survival rate of 75%. Not only are survival rates important, but most patients also have a considerable improvement in quality of life.^( 1 , 10 )^ This procedure was first performed on polycystic liver and kidney disease in 1990 and is the only definitive treatment available.^( 6 )^

Although there are numerous benefits of transplantation, there are many complications associated with the procedure, and much more when we talk about double kidney-liver transplantation. In the medical literature, many post-operative complications have been reported, the most common being infection 30%, followed by bleeding, 13%, and biliary complications 12%. The rejection rate is approximately 10%.^( 9 )^

In short-term evaluation, patients who underwent transplantation have an equivalent prognosis to other indications and, in the long term, have a better prognosis, but long-term immunosuppression was not considered.^( 3 )^ In a multicentric Spanish study, the management of immunosuppression was good in the post-operative, with only 16% presenting graft rejection and treated with optimization, and one patient needed to add corticosteroids.^( 12 )^

A study was conducted in a major German center with 36 patients who underwent liver or double kidney-liver transplantation because of polycystic disease. The results showed increased health, quality of life, and emotional and nutritional states. One patient stated that they would not undergo transplantation, and 80% would repeat the procedure.^( 13 )^

The case described stands out because of the massive hepatomegaly presented in the patient, with the liver representing over 10% of corporal weight, approximately five to six times larger than a normal-sized liver. In the medical literature, there are records of some similar cases- female patients with the liver representing 20-21% of corporal weight, all of which were treated with liver transplants.^( 7 , 14 )^

Polycystic liver kidney disease makes the transplant quite challenging. The liver and kidney volumes and the inflammatory consistency of the cysts make it difficult to access the abdominal cavity, complicating the control of perioperative bleeding.^( 5 )^ Perioperative dissection difficulty was noted in another three cases in the literature, in which liver sizes varied on an average of 7kg.^( 15 )^

Thus, the importance of knowing how to manage, indicate and manage the case of a patient with polycystic kidney and liver disease is clear because of the great impact it has had on patients’ lives. In addition to the consequences for metabolism, with renal failure and end-stage liver disease, great deterioration in the quality of life is a fundamental factor to be improved.

## CONCLUSION

It is crucial to accurately diagnose polycystic disease and determine the most effective treatment for each case, as it can significantly impact the patient’s quality of life.
